# Effects of adding poly-histidine tag on stability, antimicrobial activity and safety of recombinant buforin I expressed in periplasmic space of *Escherichia coli*

**DOI:** 10.1038/s41598-023-32782-3

**Published:** 2023-04-04

**Authors:** Sahar Roshanak, Hanieh Yarabbi, Fakhri Shahidi, Farideh Tabatabaei Yazdi, Jebraeil Movaffagh, Ali Javadmanesh

**Affiliations:** 1 Department of Food Science and Technology, Faculty of Agriculture, Ferdowsi University of Mashhad, Mashhad, Iran; 2 Department of Pharmaceutical Nanotechnology, School of Pharmacy, Mashhad University of Medical Sciences, Mashhad, Iran; 3 Department of Animal Science, Faculty of Agriculture, Ferdowsi University of Mashhad, Azadi Square, Mashhad, 9177948974 Razavi Khorasan Province Iran; 4 Industrial Biotechnology Research Group, Research Institute of Biotechnology, Ferdowsi University of Mashhad, Mashhad, Iran

**Keywords:** Computational biology and bioinformatics, Molecular biology

## Abstract

The lack of cost-effective methods for producing antimicrobial peptides has made it impossible to use their high potential as a new and powerful class of antimicrobial agents. In recent years, extensive research has been conducted to decrease the cost of recombinant proteins production through microorganisms, transgenic animals, and plants. Well-known genetic and physiological characteristics, short-term proliferation, and ease of manipulation make *E. coli* expression system a valuable host for recombinant proteins production. Expression in periplasmic space is recommended to reduce the inherently destructive behavior of antimicrobial peptides against the expressing microorganism and to decline susceptibility to proteolytic degradation. In this study, a pET-based expression system was used to express buforin I at *E. coli* periplasmic space, and its antimicrobial, hemolytic, and cell toxicity activities as well as structural stability were evaluated. The hemolysis activity and cytotoxicity of His-tagged buforin I were negligible and its antimicrobial activity did not show a significant difference compared to synthetic buforin I. In addition, in silico investigating of stability of native and His-tagged buforin I showed that RMSF, RMSD and Rg curves had followed a similar trend during 150 ns simulation. Furthermore, evaluating the modelled structures, FTIR and X-ray methods of both peptides indicated an insignificant structural difference. It was concluded that the recombinant buforin I could be a viable alternative to some currently used antibiotics by successfully expressing it in the pET-based expression system.

## Introduction

As a result of the overuse of antibiotics, antibiotic-resistant bacteria have become a global threat. Many researchers are taking an interest in antimicrobial peptides (AMP) as a response to this global challenge. Antimicrobial peptides show potential activity against mechanisms of resistance to antibiotic compounds. Because they have a great ability to kill bacterial cells^1^. Microorganisms from viruses to parasites are targeted by these agents. AMPs are interesting because they are capable of selectively disrupting bacterial cells without affecting mammalian cells^2^. In addition to the benefits of AMPs, the cost of making antimicrobial peptides is high. Using recombinant DNA technology, large quantities of these compounds can be produced at a relatively low cost^3^.

In many organisms, non-specific defense barriers are created against pathogens using cationic antimicrobial peptides (CAPs). CAPs are positively charged, and 50% of their amino acids are hydrophobic. Lysine and arginine are two basic amino acids that lead to their positive charge^4^. The rapid antimicrobial performance and resistance to adverse conditions of cationic antimicrobial peptides make them a promising new class of antimicrobial compounds^5^. The histone-derived peptide family of antimicrobial peptides do not contribute to DNA replication. The extracellular histone derivatives have strong antimicrobial properties, and the buforin family including buforin I, buforin II, buforin EC, parasin I, abhisin, hipposin, harriotin, and histonin had classified as histone-derived peptides. A purified and characterized buforin I from an Asian toad's stomach demonstrated strong antimicrobial activity against Gram-positive and Gram-negative bacteria and fungal strains^6^. Our previous studies showed that buforin I has high and board spectrum antimicrobial^6,7^, biofilm formation and biofilm radiation activities with high therapeutic index. Buforin I's safety is confirmed by the lack of cell cytotoxicity and hemolytic activity^7^. Accordingly, Cationic antimicrobial peptides like buforin I, are being developed as a promising class of antimicrobial substances^2^.

*E. coli* is the most common host for AMP expression, due to its high growth rate and known expression systems. It is nevertheless necessary to address some issues such as low yield, proteolytic degradation of hybrid protein, and toxicity of the product produced in the host^8^. To overcome these problems, some methods have been proposed, including hybrid expression, vector construction containing multiple linked genes, added anionic fragments for neutralizing cationic peptides, and periplasmic expression^9,10^.

In periplasmic expression, a peptide signal is used to direct the expressed protein to the host periplasmic space^11^ and the recombinant proteins that extracted from the periplasmic space could purify by using nickel affinity chromatography (Ni–NTA)^9^. Therefore, an affinity tag with six consecutive histidine residues is known as a poly-histidine affinity tag, or His6; it can range in length from two to ten histidine residues; must be added to the N-terminal of proteins^12^. Histidine tags have been demonstrated to facilitate the purification of proteins of interest and enhance their refolding, solubility, and stability. While fused tags have numerous advantages, they can alter the biophysical properties of recombinant proteins. There has been evidence of structural and functional differences between histidine-tagged and untagged forms of the same protein^13^.

**Figure 1:**
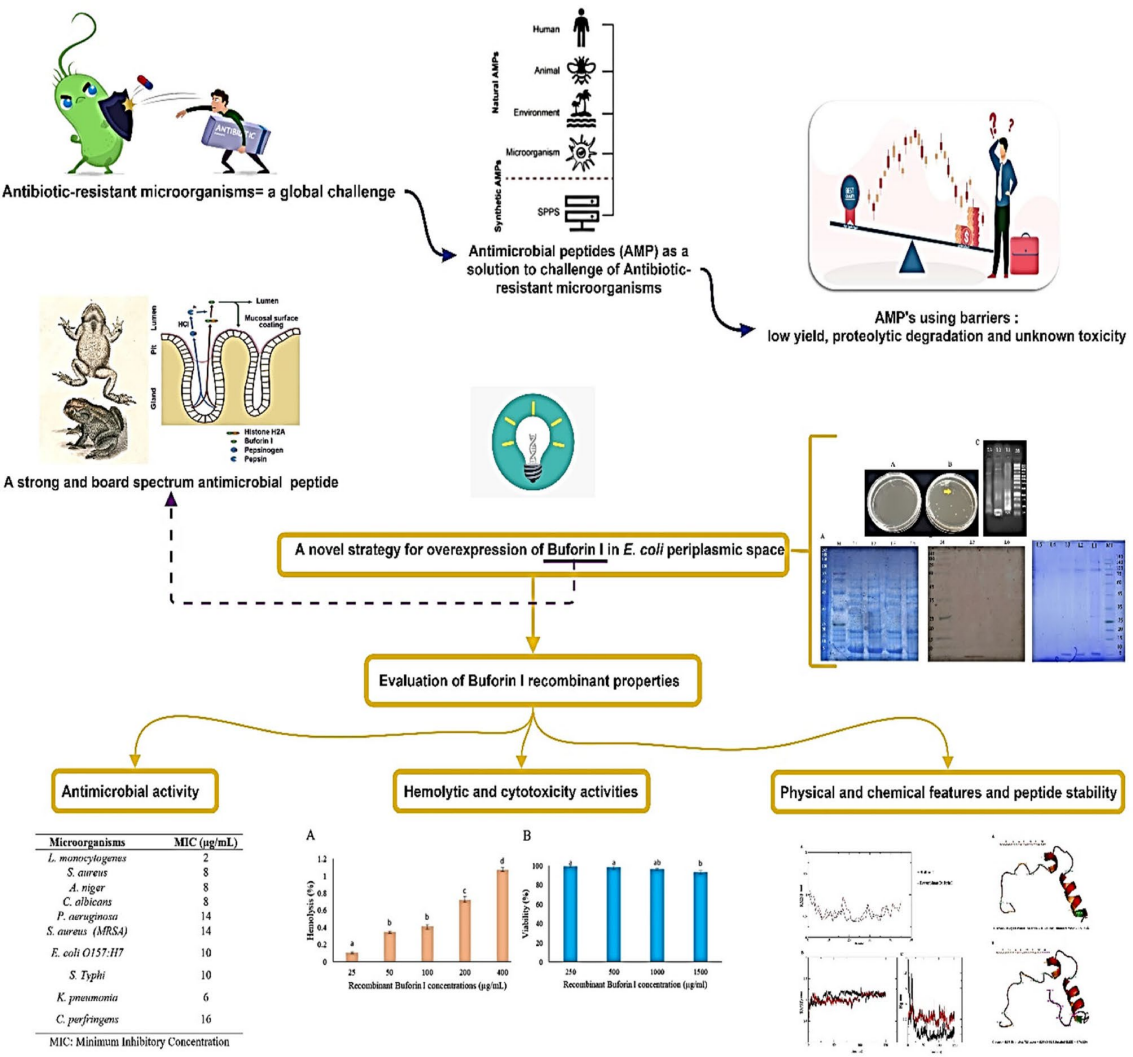
The outline of this research.

Therefore, this study was performed with the aim of recombinant production of this peptide using the method of periplasmic expression in *E. coli* BL21 (DE3) by using pET-based IPTG-inducible system and evaluating the effect of the presence of histidine-tag on antimicrobial, hemolytic and cytotoxic activities, in addition, stability and structure of recombinant buforin I. The purpose of conducting this research, the general outline of its conduct and the results obtained from it, are summarized in the Fig. 1.

## Results

### Confirmation of the presence of the gene in the plasmid

**Figure 2:**
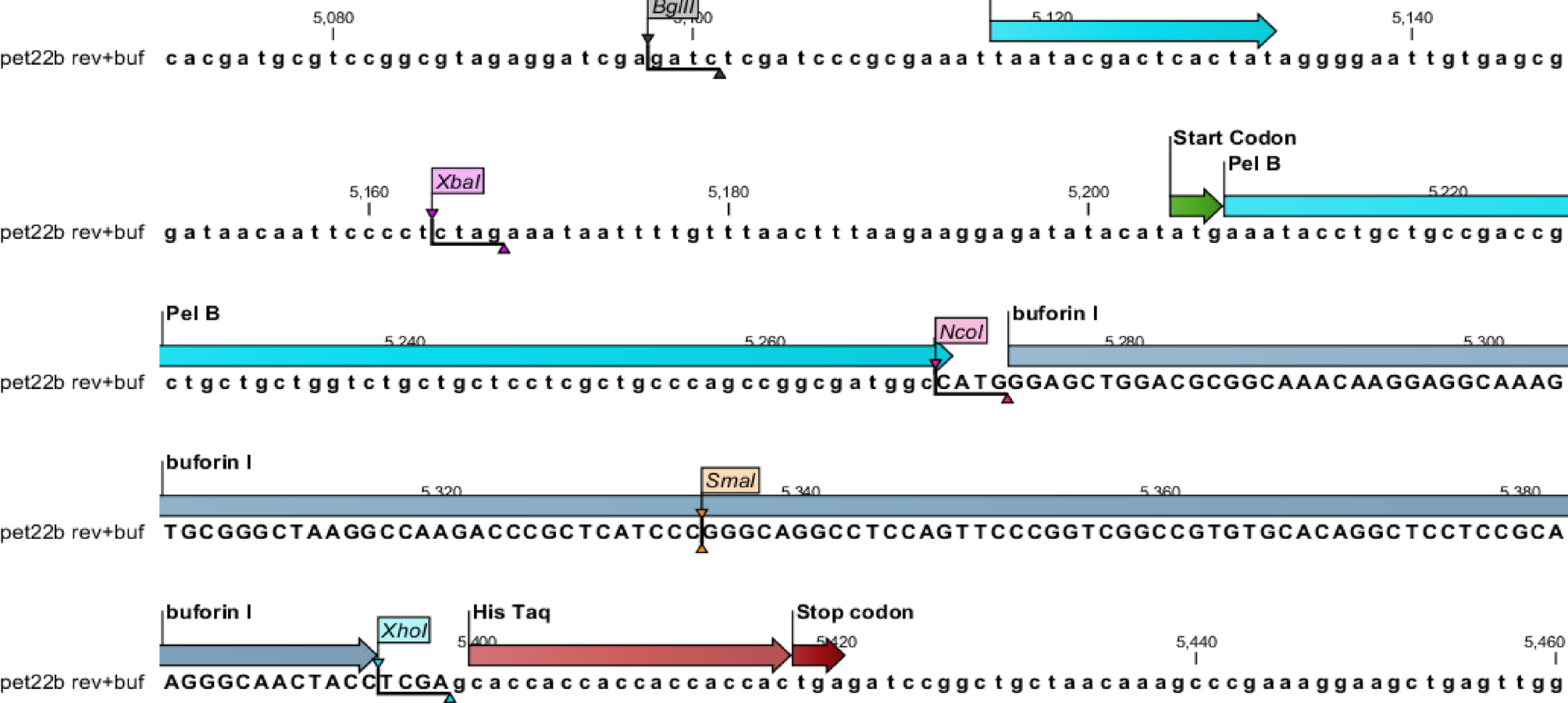
Schematic representation of the coding sequence of buforin I including pel B signal peptide (blue), buforin I sequence (gray), and His-Tag and stop codon (red).

The gene map of the expression vector pET22 b (+) was showed in Fig. 2. The buforin I DNA sequence is located between the cleavage site of *Xho*I and *Nco*I enzymes. The synthesized sequence of buforin I was inserted into the plasmid pET22 b (+) using *Xho*I and *Nco*I enzymes.

**Figure 3:**
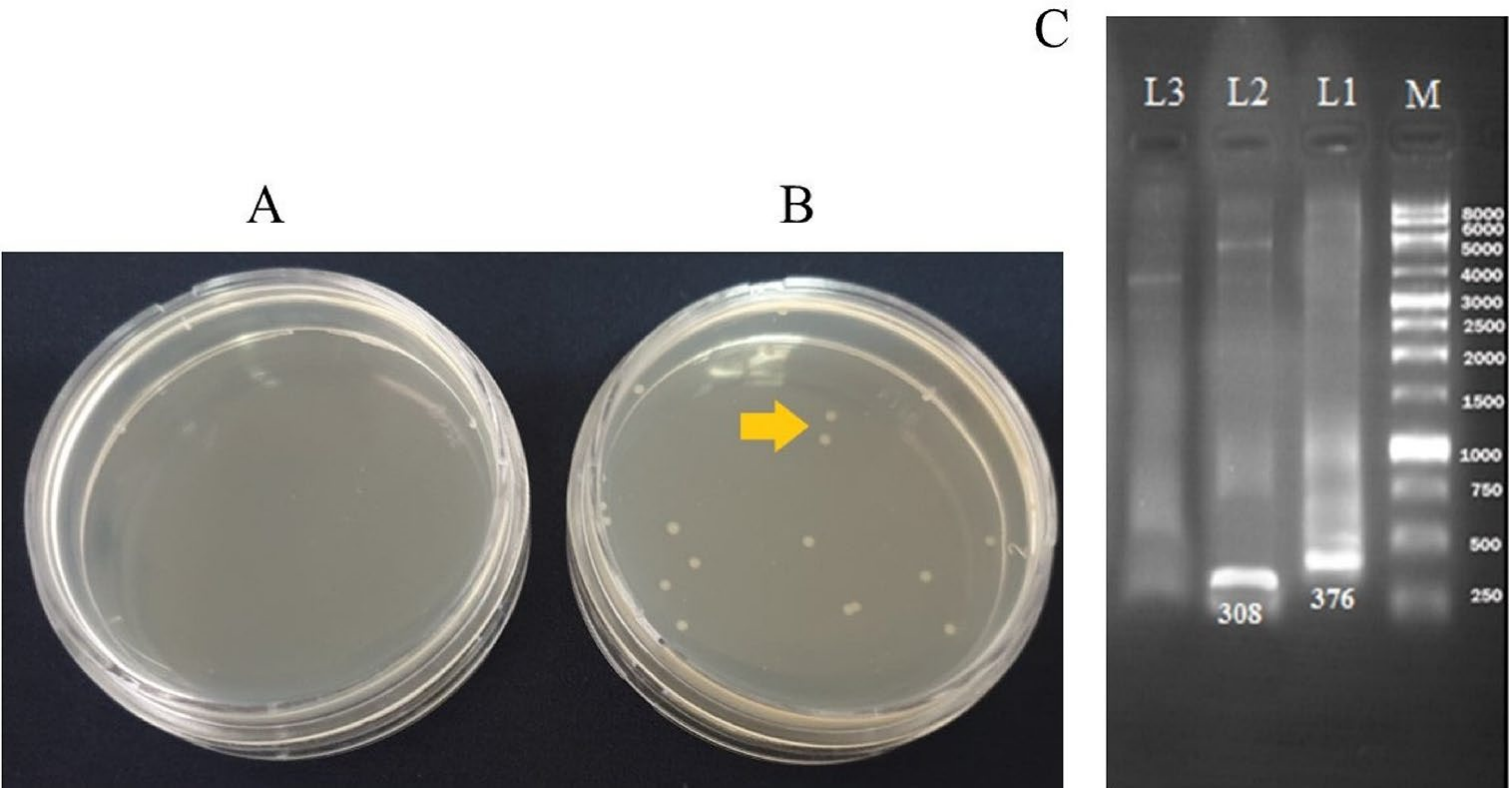
(**A**) No colony formation by bacteria without recombinant plasmid on the medium containing ampicillin, and (**B**) Colony formation (yellow arrow) by bacteria with recombinant plasmid; (**C**) Colony PCR analysis of the recombinant plasmid from *E. coli* DH5 α: M: 1 kbp DNA Ladder, L1: PCR product of *E. coli* DH5α harboring recombinant plasmid pET22 b (+)-BUF, L2: PCR product of *E. coli* DH5α harboring an intact plasmid pET22 b (+) (positive control), L3: PCR product of Plasmid-free *E. coli* DH5α (negative control).

Thus, the recombinant plasmid pET22b (+) -BUF was constructed. This recombinant plasmid was transferred to *E. coli* DH5α. Bacteria with recombinant peptides had the ability to form colonies on antibiotic-containing media (Fig. 3B), while, there was no colony formation by bacteria without recombinant plasmid on the medium containing ampicillin (Fig. 3A).

Colony PCR was performed from colonies grown on ampicillin-containing medium to confirm the presence of recombinant plasmid. Figure 3C, showed the colony PCR amplification of the transformed host strain on 1% agarose gel. In the recombinant plasmid pET22b (+)-BUF, a fragment with a length of 376 bp and in the native plasmid pET22 b (+), a fragment with a length of 308 bp were amplified. Plasmid-free *E. coli* DH5α was used as a negative control.

**Figure 4:**
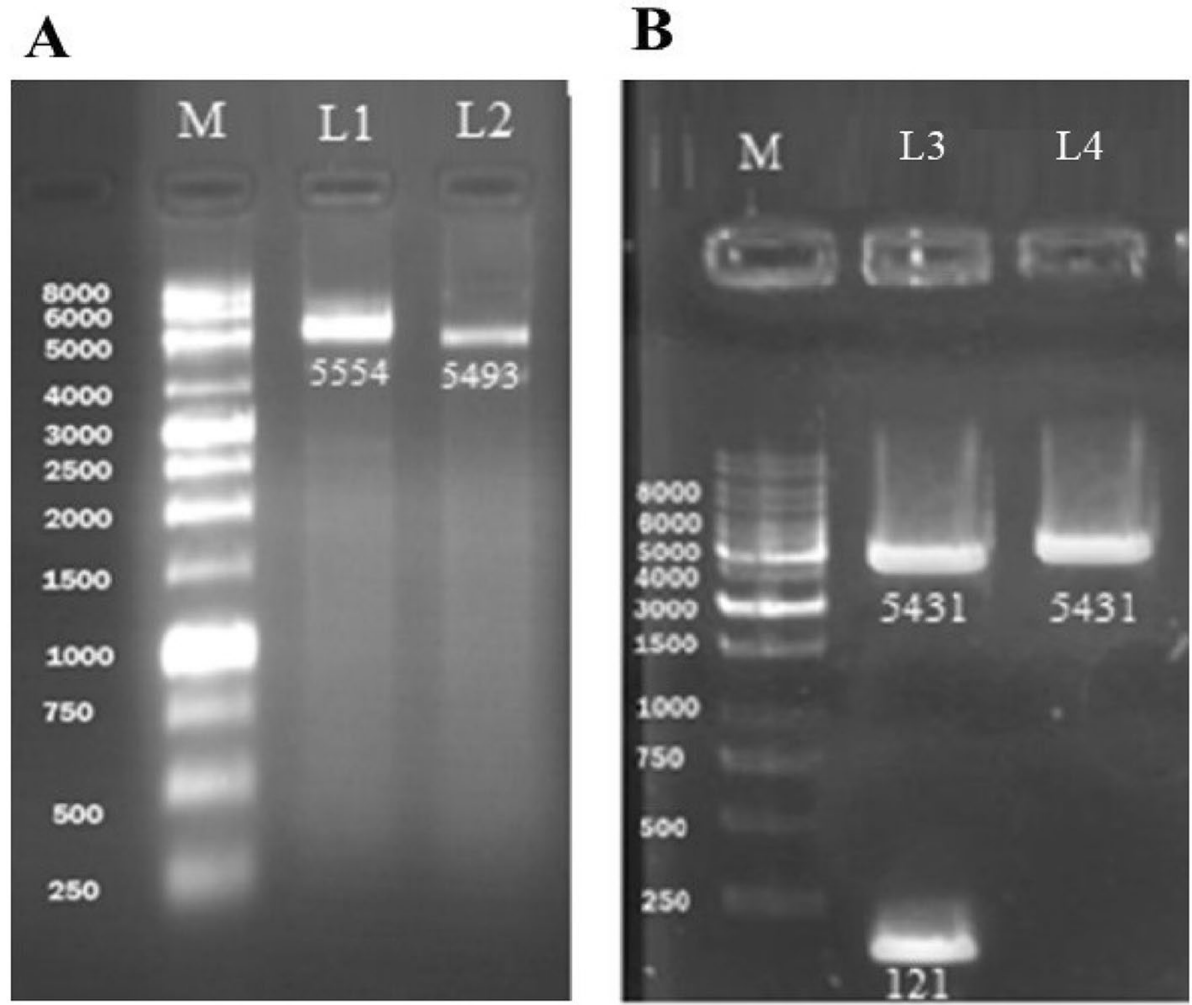
Agarose gel electrophoresis of (**A**) recombinant plasmid pET22 b (+)-BUF and plasmid pET 22b (+), and (**B**) enzymatic digestion of pET22 b ( +)-BUF and native plasmid pET 22b; M: 1 kb DNA ladder, L1: pET22 b (+) -BUF (5554 bp), L2: pET22b (+) (5493 bp), L3: digested recombinant plasmid pET22b (+) -Buforin I with *Nco*I and *Xho*I enzymes, L4: circular plasmid pET22b (+).

Plasmid extraction was performed from *E. coli* DH5α bacteria containing recombinant plasmid pET22 b (+)-BUF using a commercial plasmid extraction kit. Qualitative and quantitative plasmid content was evaluated by the nanodrop device. The recombinant plasmid concentration was 461.89 ng/µl and its adsorption ratio at 260 to 280 was 1.973. Figure 4-A showed the gel electrophoresis of recombinant plasmid pET22 b ( +)-BUF and plasmid pET 22b (+) on 1% gel. The size of these plasmids were for the recombinant plasmid pET22 b (+)-BUF and the plasmid pET 22b ( +), 5554 bp and 5493 pb, respectively, which were consistent with the results predicted with the Snap Gene software. The enzymatic digestion of the recombinant pET22 b (+)-BUF plasmid with the two enzymes *Nco*I and *Xho*I and the undigested pET22 b (+) plasmid electrophoresed and visualized on a 1% agarose gel (Fig. 4B).

### Expression of buforin I in the periplasmic space of *E. coli* BL21

**Figure 5:**
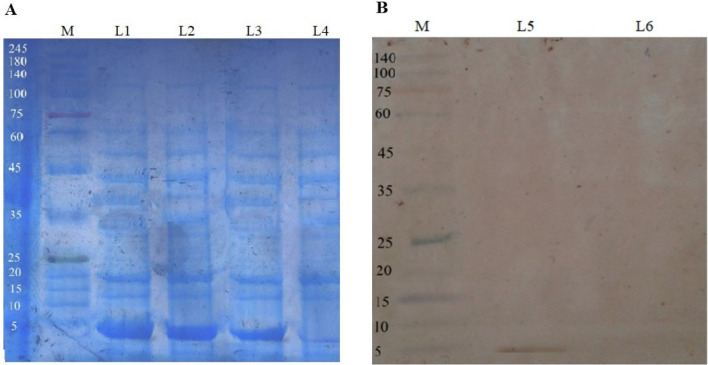
(**A**) Profile of the expressed buforin I in *E. coli* BL21 (DE3) cells at different sampling times; (**B**) Western blot analysis of the buforin I. M) protein ladder, L1) 6 h, L2) 4 h, L3) 2 h, L4) *E. coli* BL21 lacking recombinant plasmid (negative control), L5) *E. coli* BL21 cell harboring the plasmid pET22b ( +)-BUF, L6) *E. coli* BL21 harboring an intact pET22b (+) vector as the negative control.

In order to evaluate the production of recombinant protein in the expression system, the relevant samples were evaluated at 2 h intervals after the addition of IPTG on SDS-PAGE (sodium dodecyl sulfate–polyacrylamide gel electrophoresis). The profile of buforin I expression in *E. coli* BL21 at intervals of 0, 2, 4, and 6 h after the addition of IPTG is showed in Fig. 5A. There was a band approximately with the size of 5 kDa, whose intensity increases from zero to 6 h. Figure 4B, shows the result of the western blot test. The band with the size of 5 kDa on the nitrocellulose paper confirmed the expression of buforin I in *E. coli* BL21 (DE) containing the recombinant plasmid pET22b (+)-BUF (Fig. 5B). The brown color band formed on nitrocellulose paper is due to the enzymatic reaction of DAB with the enzyme superoxide dismutase, which binds to the anti-histogram monoclonal antibody (Images of agarose gels, SDS-PAGE, and Western blot can be accessed separately as supplementary information).

### Purification and quantification of buforin I

**Figure 6:**
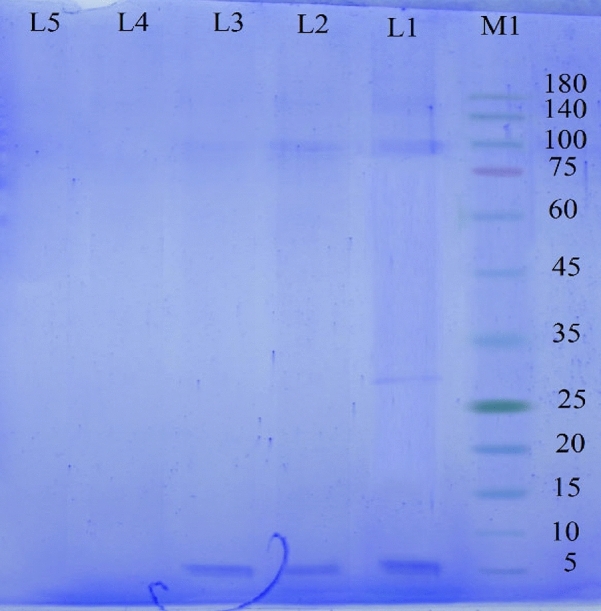
SDS-PAGE of purified recombinant buforin I using Ni–NTA column: M) protein marker, L1) Elution 1, L2) Elution 2, L3) Elution 3, L4) Elution 4 and L5) negative control (*E. coli* BL21 without recombinant plasmid).

**Figure 7:**
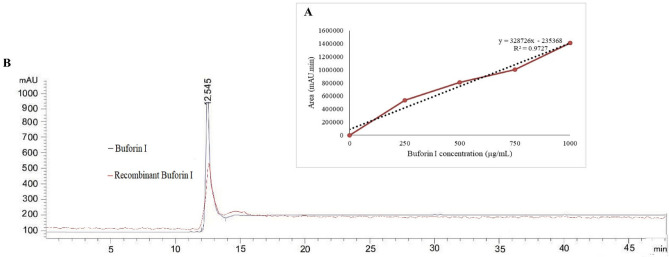
(**A**) Calibration curve with a R-squared value of 0.97 (dashed line) for the buforin I concentration range of 0–1000 µg/mL (data points are given as filled circles); (**B**) Analytical HPLC of recombinant and synthetic buforin I indicating purity of recombinant buforin.

Research has shown that the tendency of the amino acid histidine to bind to the nickel metal is high. Protein purification is possible by adding 6 to 8 histidines at the carboxyl or amino terminus of the protein^12^. As shown in Fig. 6, from Elution 1 to Elution 4, the intensity of the background bands is reduced. Therefore, the purity of the recombinant protein was considerably high. According to the protein marker, the molecular weight of the purified recombinant protein was about 5 kDa. The production efficiency of buforin I peptide in this study using Bradford method was estimated around 0.327 µg/mL. Elution 3 containing the recombinant buforin I was freeze-dried and stored at − 20 °C for next experiments. In addition, the result of HPLC analysis showed that the purity of recombinant buforin I was 94.64% and confirmed its authenticity and integrity (Fig. 7).

### Evaluation of antimicrobial activity, hemolytic and cytotoxicity activities and therapeutic index

Table 1 showed the results of the antimicrobial effect of recombinant buforin I by MIC assay.

**Table 1:** MIC values of recombinant buforin I against some pathogenic bacterial and fungal strains.

Microorganisms	MIC (µg/mL)
*L. monocytogenes*	2
*S. aureus*	8
*A. niger*	8
*C. albicans*	8
*P. aeruginosa*	14
*S. aureus (MRSA)*	14
*E. coli O157:H7*	10
*S. Typhi*	10
*K. pneumonia*	6
*C. perfringens*	16

MIC, Minimum Inhibitory Concentration.

**Figure 8:**
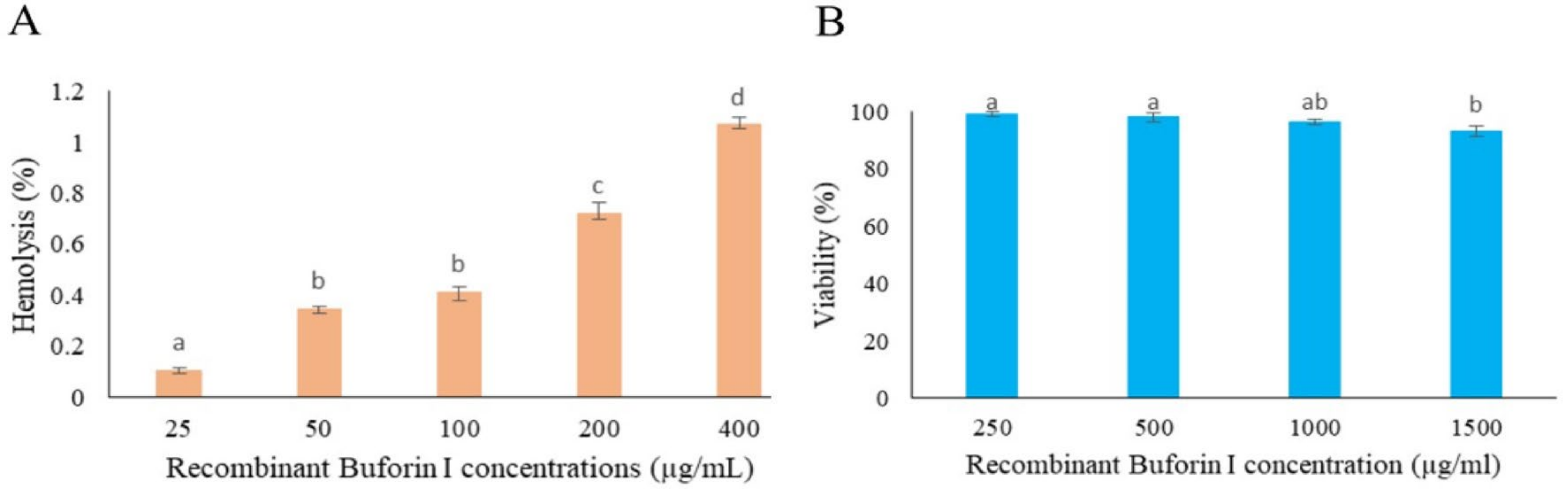
(**A**) Evaluation of hemolytic and (**B**) cytotoxic effects of recombinant buforin I on human blood hemoglobin and human fibroblast cell line, respectively. Different lowercase letters indicate significant difference (*p* < 0.05).

Hemolytic and cytotoxicity activities of buforin I, showed a negligible hemolytic effect or toxicity (Fig. 8). As GM of MIC against tested microbial strains, and MHC were 8.02 µg/mL and 800 µg/mL, respectively, calculated therapeutic index for recombinant buforin I was 97.54. This value was significantly higher than the value obtained for buforin I (76.73) indicating the greater effectiveness of recombinant buforin I.

### In silico investigation of physical and chemical features and peptide stability

According to the results of investigation the features of buforin I after adding 6 histidine tags compared with native buforin I which were presented in Table 2, there were not significant differences in PI, net charge, half-life and solubility of both peptides. Amino acid composition of buforin I and recombinant buforin is showed in Table 3.

**Table 2:** Physical and chemical features of buforin I and recombinant buforin I by Protparam.

Peptide	Amino acid length	M.W(Da)	PI	Net charge	Gravity	AI	Instability index	HL (h)	Solubility
Buforin I	39	4262.99	12.41	11.9	− 0.992	62.56	46.13	> 10	0.761
Recombinant buforin I	45	5085.84	12.41	12	− 1.287	54.22	44.05	> 10	0.710

MW, Molecular weight; AI, Aliphatic index; PI, isoelectric point; Net charge, Charge at pH = 7.4 (cytoplasm); HL, half-life considered in *E. coli*, in vivo; h, hours.

**Table 3:** Amino acid composition of buforin I and recombinant buforin I by Protparam.

Peptide	Amino acid composition (%)													
	Ala (A)	Arg (R)	Asn (N)	Gln (Q)	Gly (G)	His (H)	Leu (L)	Lys (K)	Phe (F)	Pro (P)	Ser (S)	Thr (T)	Tyr (Y)	Val (V)
Buforin I	10.3	17.9	2.6	5.1	17.9	2.6	7.7	12.8	2.6	2.6	5.1	2.6	2.6	7.7
Recombinant buforin I	8.9	15.6	2.2	4.4	15.6	15.6	6.7	11.1	2.2	2.2	4.4	2.2	2.2	6.7

**Figure 9:**
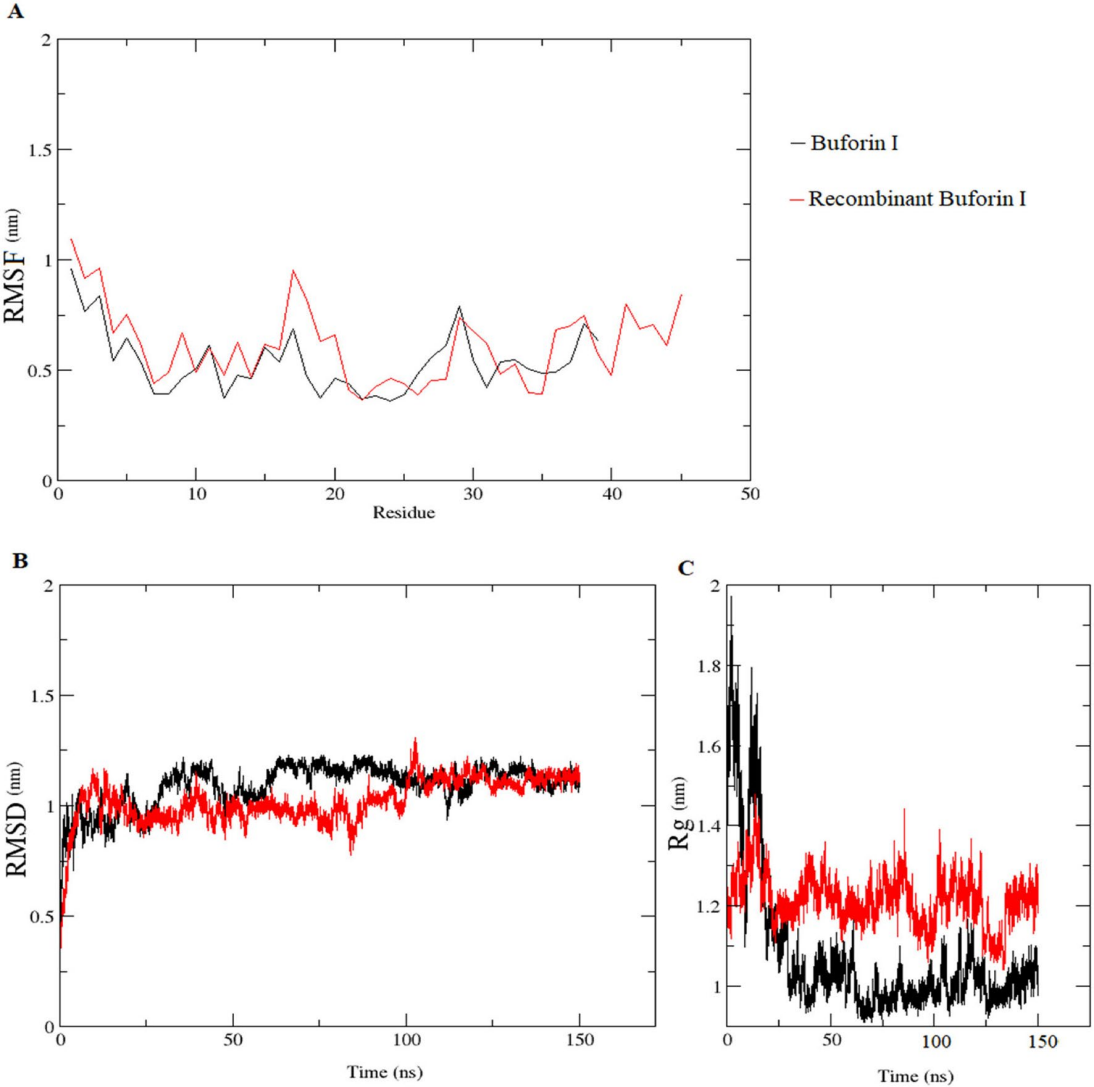
In silico assessment of stability of buforin I and recombinant buforin I at 310 °K. (**A**) RMSF, (**B**) RMSD, and (**C**) Radius gyration.

The results of in silico stability investigation of recombinant buforin I and buforin I were presented in Fig. 8. The dynamic behavior of individual amino acid residue of peptides was analyzed using the RMSF value (Fig. 9A). The similarity was observed in pattern of RMSF curves for native buforin I (black curve) and recombinant buforin I (red curve). The RMSD is an assessment of how much the structure of a peptide changes over time in comparison to its initial structure. By increasing his tags, the RMSD patterns did not deviate drastically over 50,000 ns for both peptides (Fig. 9B). Peptide compactness is measured by the radius of gyration (Rg). The plot of Rg was illustrated for alpha-carbon atoms vs time which is depicted in Fig. 9C. Bothe buforin I and recombinant buforin I maintained their structural compactness during simulation. The insignificant increase in the Rg score confirms the increase in polarity and hydrophilicity (more negative GRAVITY and lower Aliphatic index), also decrease in the globularity of recombinant buforin I in comparison with native buforin I.

### In vitro peptide stability investigation

**Figure 10:**
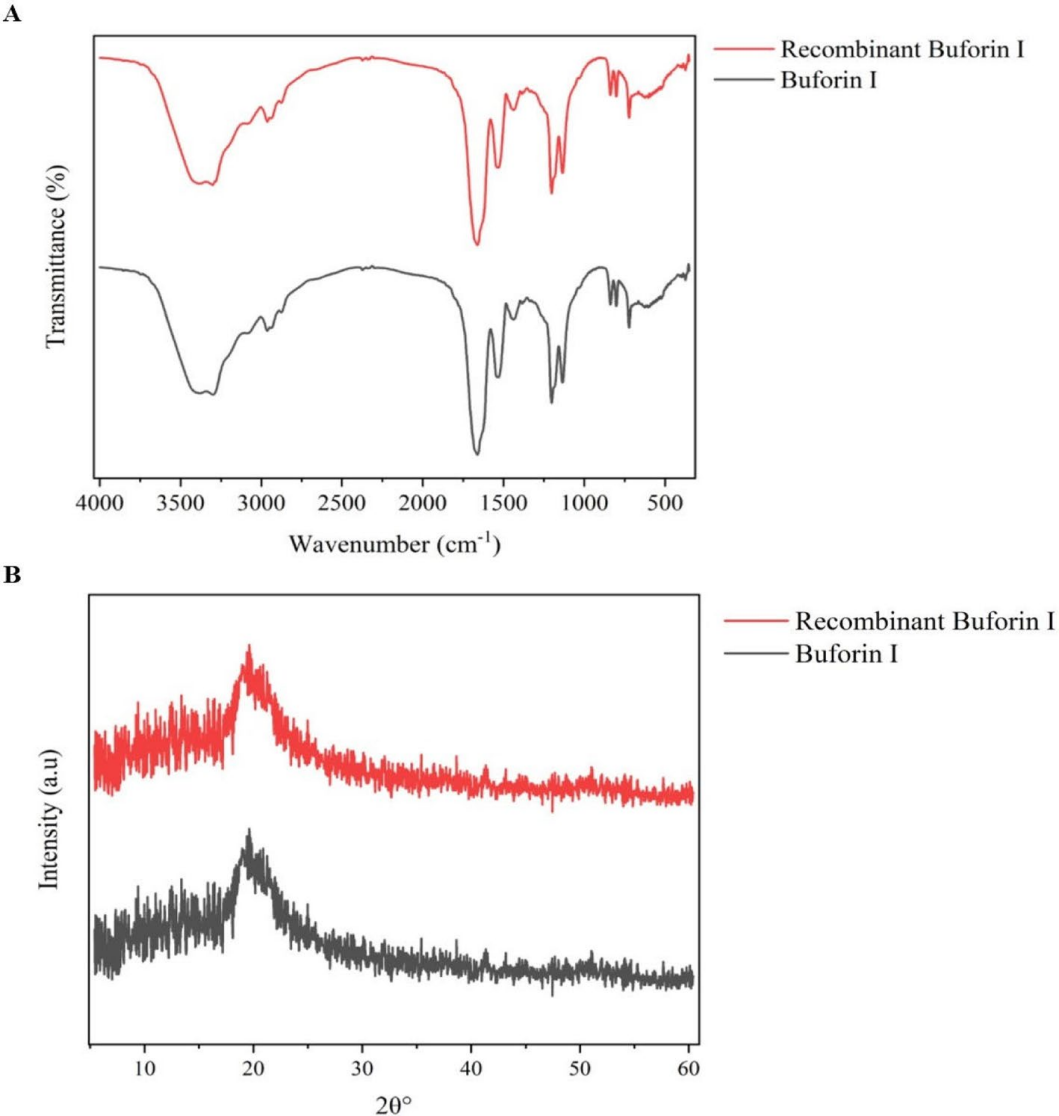
In vitro assessment of stability of buforin I and recombinant buforin I. (**A**) FTIR, (**B**) XRD.

As shown in Fig. 10A, buforin I and its recombinant had similar peaks and suggesting that the His tag has no detectable effect on the secondary structure of buforin I. The asymmetric stretching frequencies of the amide I band (1600–1700 cm^−1^), and N–H band (3200–3500 cm^−1^) in the FTIR profile reveals the secondary structure of a peptides. Both peptides showed maxima in the Amide I band 1655 cm^−1^, which is generally assigned to α-helix conformation^14^. This absorption had the corresponding band in the amide II region at 1538 cm^−1^. In the amide III region, the 1205 cm^−1^ and 1136 cm^−1^ peaks confirmed the presence of α-helix and β-turn conformers. The β-turn could be associated with proline present in amino acid sequences which most important amino acid in change chain’s direction and creating loop structure, as a secondary-structural motif in globular proteins^15^.

To obtain further information on the internal structure, XRD diffraction was performed. In the XRD measurement, both peptides showed a broad band at 2θ = 20° (Fig. 10B). By using the Bragg equation:

$$2{\text{d}}\sin \theta = \lambda$$

where d is the d-spacing, 2θ is the scattering angle and λ is the X-ray wavelength^16^; the corresponding d ≈ 4.43 Å indicated that the distance between two stacking α-helical structures which attached with a proline hinge region is ≈4.43 Å.

## Discussion

With attributes such as high specificity, potency, tissue penetrability, low immunogenicity, and tissue accumulation, many peptide-based drugs have been successfully approved by the FDA. Increased antibiotic resistance in bacteria has brought the research and development of AMPs and their use as a promising alternative antimicrobial strategy into focus. Therefore, upscaling AMP production is critical to their use. AMP activity, peptide length and in some cases its structure can complicate production and often require optimization of production through trial and error or new strategies. Production methods and strategies have emerged that focus on both molecular design and the use of recombinant expression in a variety of species^17^. Strategies for improving antimicrobial peptide production discussed by Deo et al.^17^, are summarized in Table 4.

**Table 4:** The advantages and disadvantages of different synthesis and expression strategies for AMPs^17^.

Strategies for improving antimicrobial peptide production			
Production of AMPs	Method	Advantages	Disadvantages
Non-ribosomal peptide synthesis	solid-phase-peptide synthesis	• No proteases during production • Comparable or improved activity • High purity • The synthesis of libraries of new AMPs in one experimental method	• High costs • Select peptides difficult to synthesize
	Synthesis by using D-peptides	• The enantiomeric form of amino acids • Increase stabilityand protection against degradation	• Is still costly when compared to production in bacterial systems • Probable of secondary structure disruption and potentially cause a loss of activity of the respective protein
Ribosomal protein synthesis—expression and purification of AMPs using heterologous systems	Bacterial expression systems	• High purity • High expression levels • Low costs • Well established method	• Prone to protease degradation • Endotoxin contaminants • No post-translational modifications
	Fungal expression systems	• Efficient secretion if utilized • Post-translational modifications • High expression levels • Low cost	• Fermentation production • Hyperglycosylation may occur
	Plant based expression systems	• Large scale production • Low cost • Post-translational Modifications • Option for cell suspension	• Genetic modification is difficult • Long growth time • Low yields and low stability
	Insect based expression systems	• Genomic or plasmid expression • Post-translational modifications	• Low yields • High cost • Difficult to upscale • Potential issues with Lytic cycle
Fusion-protein based approaches for recombinant production of AMPs	Fusion-protein based secretion	• Improved purification • Improved peptide stability • Decreased toxicity	• Degradation during purification • Potential folding issues • Potential toxicity
	Fusion-protein based production using inclusion bodies	• Easy purification • Decreased cytotoxicity	• Lower yields • Protein misfolding • No post- translational modifications
	Fusion-protein based enhanced solubility	• Higher intracellular concentrations • Less protein misfolding • Improved production method	• Low expression yields • High toxicity in the cell
	Fusion-protein based masking of AMP toxicity	•Higher yields • Low production costs • Prevents inclusion body formation	• Some strategies cause protein to enter inclusion bodies
	Hybridization expression of AMPs	• Potential novel AMPs • Coproduction of two AMPs • Decreased cytotoxicity • Improved yields, selectivity, activity, and stability	• Inactive hybrids • Need for characterization of hybrid AMPs
	Cleaving the fusion-protein	• Ability to use autocleavage • Removal of fusion proteins after use • High yields • Used with other methods	• Extra purification steps • Dependent on protein solubility • Dependent on cleavage site Accessibility
Multimeric expression		• Improved yields • Improved stability	• Expression based on copy number cannot be predicted • Expression system can affect expression

AMPs, Antimicrobial peptides.

Periplasmic expression of proteins has several important advantages over intracellular expression: (1) toxic or lethal effects of some target proteins can be reduced or eliminated through periplasmic expression; (2) by expressed periplasmically or extracellularly, target proteins can become more solubilized ; (3) an unwanted primary methionine in a protein sequence that it would not normally contain can be prevented by using signal peptides in periplasmic or extracellular expression; and (4) protein secretion in the periplasmic environment facilitates the process of downstream processes^18,19^.

To date, no study has been performed on the production of recombinant buforin I, but there are few studies on the recombinant production of buforin II. Pyo et al. (2004) and Wang et al. (2011) produced buforin II in the *E. coli* expression host by fusion protein assay. Production yields in these two studies were 1.24 g of pure buforin II per 10 L of cultured cells and 3.1 mg/l, respectively. Jang et al. (2009) developed buforin II, parasin I, and pexiganan as a conjugated dual systronic expression system. They reported that the production efficiency of each of these peptides was about 100 mg/l of *E. coli* culture. Tanhaiean et al. (2018) investigated the periplasmic expression of chimera lactoferrin (ID: NP_001290496.1) incorporated into a part of camel lactoferrin (ID: AHJ37525) using plasmid pET22 b (+) in the *E. coli* BL 21. Their results showed the success of using this expression system. The recombinant peptide had significant antimicrobial potency. The production efficiency of the recombinant peptide was 0.42 g /L.

One of the most important mechanisms to control gene expression in all organisms is mRNA degradation^20^. This mechanism makes it possible for cells to adapt to their surroundings by responding to environmental signals. The half-life of an mRNA in *E. coli* varies from 2 to 4 min for a typical mRNA to 17 min for an ompA (outer membrane protein) mRNA^21^. For most mRNAs, the degradation process begins with the endonucleolytic cleavage. The major endonucleases known to degrade mRNAs in *E. coli* are most often RNase E and rarely RNase III^22^. Following the action of these two non-selective ′3-exonuclease enzymes, the polynucleotide phosphorylase and RNase II lead to mRNA degradation. Substrates with ′5-monophosphate terminus are detected by the RNase E enzyme detection portion^23^. The formation of the hairpin structure at the ′5 end of an mRNA causes its faster uptake into the cell translation machine. It also leads to higher stability of the mRNA against exonuclease activity and thus increases its half-life^24^. Sletta et al. (2007) showed that by adding the signal sequence of pel B, ompA and CSP peptides to the beginning of the sequence of three important therapeutic proteins Granulocyte–macrophage Colony-stimulating Factor (GM-CSF), Interferon alpha 2b (IFN-2b) and single-chain antibody variable fragment (scFv-phOx), the expression level of GM-CSF and scFv-phOx in *E. coli* was 1.7 and 2.3 g/l, respectively, while IFN-2b expression increased. These results showed that adding the peptide signal sequence to the beginning of the target protein gene sequence could not only direct it to the periplasmic space, but also stimulate mRNA translation. In fact, placement of the signal peptide at the beginning and periplasmic expression of the target protein sequence increases the mRNA half-life, which in turn increases the expression efficiency^20^.

The results of this study showed that the production of buforin I in the periplasmic space of the expression host, *E. coli* BL21, showed acceptable efficiency (yield = 0.327 µg/mL) using pel B as signal peptide and pET22b (+)vector. Therefore, this method could be used as a method to reduce the production costs of this powerful antimicrobial peptide.

In our previous study, the antimicrobial effect of native buforin I was assessed against same microbial strains (MIC for *L. monocytogenes, S. aureus, A. niger, C. albicans, P. aeruginosa, S. aureus* (MRSA), *E. coli* O157:H7, *S. Typhi, K. pneumonia* and *C. perfringens* was 4, 10, 10, 10, 12, 14, 14, 14, 6, and 16 (µg/mL) of buforin I, respectively)^7^. As the difference in MICs value for the buforin I and recombinant buforin I for all microbial strains were less than two-fold, the sensitivity levels were considered equal, therefore, there is no significant difference between the antimicrobial effect of recombinant buforin I and synthetic buforin I. This decrease in MIC value can be considered due to the increase in the positive charge of the recombinant buforin I peptide by the addition of the histidine tag^25^. Despite hemolytic and cytotoxicity activities enhancing by increasing buforin I concentration, this peptide did not exhibit negligible hemolytic effect or toxicity. These results were consistent with our previous study when the peptide's hemolytic and cytotoxicity activities were evaluated at similar concentrations and by similar methods^7^.

The in silico approaches, such as bioinformatics and data analysis, have been widely used to study peptide behavior in recombinant peptide expression and purification^11^. The instability index indicates the peptide stability *in-vivo* as well as *in-vitro*. An unstable peptide has an instability index of > 40, while a stable peptide has an index of < 40^26^; then both native and recombinant buforin I considered as unstable. As well as instability index, aliphatic index (AI) also be used to determine peptide stability. A protein's AI can be expressed as the relative volume captured by its aliphatic side chains (Alanine, Valine, Leucine, Isoleucine). Earlier a good correlation was established between AI and thermo-stability of proteins; having a high AI value indicates that the protein is stable under a wide range of temperatures and has a more flexible structure^26^. AI for recombinant buforin I and buforin I were 54.22 and 62.56, respectively, the difference were caused by a change in amino acid composition and a decrease in alanine, valine, and leucine percentages in recombinant buforin I. Moreover, peptide hydrophobicity or hydrophilicity was evaluated by the GRAVY score. For each protein, the GRAVY score was determined by dividing the sum of hydropathy values of all amino acids by the number of residues. This score ranges from − 2 to + 2, with a negative score indicating hydrophilicity and a positive score indicating hydrophobicity. Proteins with more negative GRAVY score are considered to be hydrophilic in nature with good solubility and vice-versa^26^. GRAVY score of recombinant buforin I was more negative that showed increasing the polarity of peptide in result of adding His tags.

**Figure 11:**
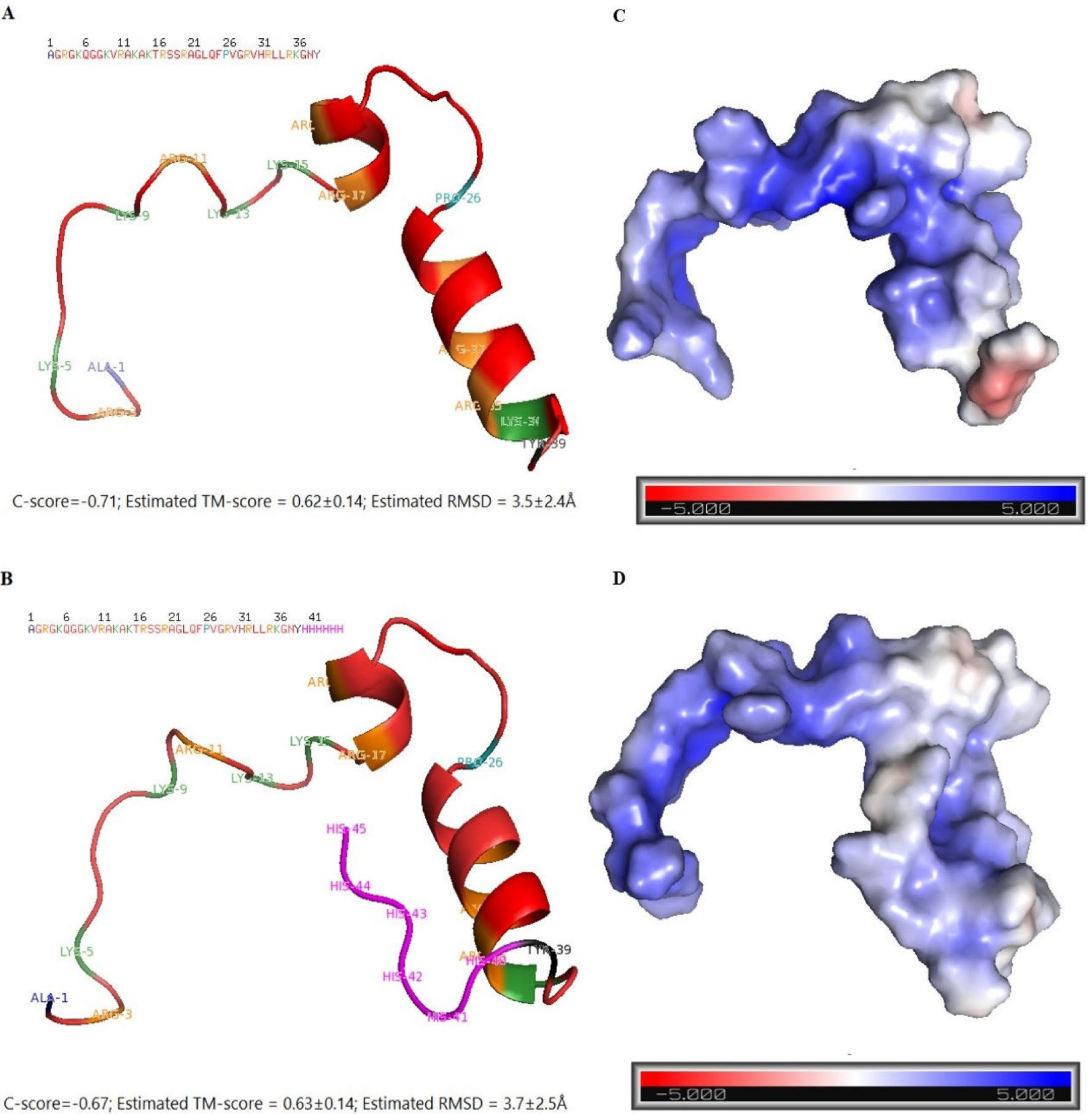
The predicted structures of (**A**) buforin I and (**B**) recombinant buforin I modeled by I-TASSER; (**C**) buforin I and (**D**) recombinant buforin I molecular electrostatic potentials.

In addition of high positive charge of buforin family which facilitates the binding of these molecules to negatively charged extracellular and intracellular targets, presence of proline cause to create hinge-like structure which is necessary for the translocating these molecules from the cell membrane without disrupting the membrane's structure^27^. Proline was found to stabilize the a-helical conformation^28^, as illustrate in Fig. 11A and B, as a rigid amino acid is located in the 26th position and lead to helix structure in both native and recombinant buforin I. It should be noted that the difference between C- score, TM-score and RMSD for native buforin I and recombinant buforin I wasn’t significant. C-score is a confidence score for estimating the quality of predicted models by I-TASSER. It is calculated based on the significance of threading template alignments and the convergence parameters of the structure assembly simulations. The TM-score and RMSD are known standards for measuring structural similarity between two structures which are usually used to measure the accuracy of structure modeling^29^.These results indicate that increasing the His tag had no effect on the structure and subsequent antimicrobial activity of buforin I. Furthermore, showed the importance of peptide positively charged residues for the initial binding to negatively charged vesicles.

The results of the structure stability evaluation of peptides by XRD and FTIR also confirmed above issues. FTIR spectroscopy detects the vibrations in a molecule. Proteins/peptides contain groups with IR active vibrations such as the amide group (i.e., O=C–NH), αC-H, and residue groups of amino acids. These groups result in several intensive bands in the FTIR spectra. The amide I band around 3300 cm^-1^ is from the N–H stretching vibration in the amide group. The amide I band (mainly from the stretching mode of C=O in the amide group), amide II bands (from the bending mode vibration of N–H in amide group), and amide III (CN stretching, NH bending) are in the range of 1600–1700 cm^−1^, 1500–1560 cm^−1^, and below 1400 cm^−1^, respectively^14^. Among all the amide bands, the position of the amide I band is most sensitive to the conformation (i.e., the secondary structures) change of proteins/peptides and has been widely used to evaluate the fraction of various secondary structures in a protein/peptide^30^. The result of this study showed α-helical structure of native buforin I (maxima in the Amide I band 1655 cm^−1^) was not affected by adding His-taq to the C-terminal of its amino acid sequence. In addition, the results of the XRD test confirmed the special function of the amino acid proline in creating a hinge in the helical structures of both peptides.

There are physical forces such as complementarities between protein surfaces, residue interface propensities, conformational changes, hydrogen bond and van der Waals interactions involved in the physical interactions of proteins, but the electrostatic potentials of proteins could serve as a major basis for their physical interactions. Electrostatic potentials play a major role in their specific physical interactions^31^. As shown in Fig. 11C and D, change red color in the electrostatic potential of the C-terminal surface of buforin I to blue color in the electrostatic potential of the C-terminal surface of recombinant buforin I indicated the increasing the positive electrostatic potentials that consistent with of the His-taq.

## Conclusion

The favorable properties of buforin I have led to its use as an alternative to antibiotics in the production of animal, animal, and human infections. Its properties included: thermal stability and stability in 50% human plasma, antimicrobial activity, inhibition of microbial growth for a significant period of time, inhibition of biofilm formation and biofilm destruction, lack of hemolytic activity and cytotoxicity, as well as high therapeutic index. This peptide can also be used as a preservative in the food industry or to prevent the formation and destruction of biofilms in food production lines, medicine and dentistry. However, cationic antimicrobial peptides, such as buforin I, had potential benefits such as broad-spectrum antimicrobial, non-selective performance, thermal stability, and resistance to pH changes. However, their application is limited to two important factors: cost and production efficiency and their sensitivity to proteolytic degradation. In the present study, we tried to introduce an optimal production method by evaluating the feasibility of periplasmic expression of this peptide in *E. coli*. The results of this study showed a yield of 0.375 mg/mL of recombinant buforin I in the periplasmic space of this bacterium.

## Materials and methods

### Bacterial strains, vectors and other regents

In this experiment, plasmid pET22 b (+) (5493 bp, Novagene, USA) was used as the expression vector. Also, *E. coli* strains DH5 α and BL21 (DE3) (Iranian Biological Resources Center) were used as hosts for cloning (amplification) and expression of recombinant peptide, respectively. *E. coli* BL21 (DE3) has the λ prophage carrying the T7 polymerase that for protein over-expression needed T7 promoters. The recombinant protein expression was induced by IPTG (Sigma, Country) under the control of a strong T7 promoter in *E. coli* BL21 (DE3). Ampicillin was used to select the transformed colonies on the Luria–Bertani (LB) agar (Miller). The Luria–Bertani (LB) medium (Miller) was used for bacterial culture. A pre-equilibrated Ni–NTA affinity column (Bio-Rad) and imidazole (AppliChem, Spain) were used to Purification of recombinant protein. 1 kb DNA Ladder and 245 kDa protein Ladder, and *Nco*I and *Xho*I Restriction enzymes were purchased from Roche (Germany) and Thermo Fisher Scientific (USA) companies, respectively.

### Gene synthesis and cloning

The amino acid sequence of buforin I (AGRGKQGGKVRAKAKTRSSRAGLQFPVGRVHRLLRKGNY) was provided from the national center of biotechnology information (NCBI) database (https://www.ncbi.nlm.nih.gov/) with accession number: P55897. Nucleotide sequence obtained by using CLC Main Workbench software version 5.5 (GCTGGACGCGGCAAACAAGGAGGCAAAGTGCGGGCTAAGGCCAAGACCCGCTCATCCCGGGCAGGCCTCCAGTTCCCGGTCGGCCGTGTGCACAGGCTCCTCCGCAAGGGCAACTAC). A 121 bp DNA fragment of the gene was sent for synthesis by Genscript (USA), after optimizing the codons to increase the expression level. The buforin I gene fragment tagged with hexa-histidine at the N-terminus was ligated into the pET22 b (+) expression vector. The recombinant plasmid which contained the target gene encoding sequence was transformed into *E. coli* DH5α competent cells for amplification. The transformation was performed using the heat shock and based on a standard protocol^32^.

Two µl of recombinant plasmid was added to 50 μl of competent bacteria, incubated on ice for half an hour. It was placed in a hot water bath at 42 °C for 60 s. Then 200 µg of SOC medium was added and incubated at 37 °C in the shaker incubator. From the contents of the microtubule, a linear culture was given on the LB agar containing ampicillin (100 μg/ml) and incubated overnight at 37 °C.

The positive clones were screened by colony PCR and sequencing. Set up 20 μl reactions as follows: 10 μl of 2X Master Mix (1.5 M contains dNTPs, MgCl2, buffer components and stabilizers) (Amplicon, Denmark), 1 µl T7 forward and reverse primers (200 nM), 1 µl of template DNA (150 ng) and 8 µl sterile deionized water. The thermal program for PCR reaction was performed using the Biometra T-personal thermocycler (Biometra, Germany) as follows: Initial denaturation for 2 min at 94 °C, 30 cycles of denaturation of the double-stranded DNA at 94 °C for 60 s, annealing at 61 °C for 45 s and Extension of primers with a *Taq* DNA polymerase at 72 °C for 45 s, then one cycle of final Extension for 10 min at 72 °C^33^.

The sequence of T7 promoter primers is as follows:

- 3′ AATACGACTCACTATAG.
- 5′ GCTAGTTATTGCTCAGCGG.

The recombinant plasmid was extracted using a kit from QIAGEN, Germany, and it was purified with the miniprep plasmid purification kit (Sangon Biotech, China). After extraction, plasmid DNA was concentrated via ethanol precipitation^32^. To confirm the presence of the target gene, the recombinant plasmid was double digested with enzymes *Xho*I and *Nco*I. After incubating the digestion reaction at 37 °C for 2 h, the product of double digestion was electrophoresed on a 1% agarose gel. A NanoDrop® ND-1000, USA device was used to determine the concentration of recombinant plasmid DNA. The plasmid containing buforin I gene was transformed into competent *E. coli* BL21 (DE3) using the heat shock and based on a standard protocol^32^.

### Expression and extraction of recombinant peptide

A single colony was inoculated into 5 ml of LB Agar medium containing 100 μg/mL ampicillin and incubated overnight at 37 °C and 120 rpm. Then, 250 μL of LB broth containing recombinant *E. coli* BL21 (DE3) was added to 25 ml of autoclaved LB medium. After the OD600 culture reached 0.6, protein expression was induced by 1 mM IPTG and incubated for 6 h at 37 °C and 120 rpm. Sampling was performed every hour from the culture. Then, samples were centrifuged at 5000 rpm for 5 min at 4 °C and the cell pellet was collected. It was sonicated at 25 W for 30 s, after resuspension in lysis buffer (20 mM NaH_2_PO_4_, 25 mM imidazole and 0.5 M NaCl, pH = 7.4)^32^. after which the cell debris was removed by centrifugation at 4 °C at 13000 × *g* for 10 min, the soluble crude extract was electrophoresed by SDS-PAGE on 12% separating and 5% stacking polyacrylamide gels and Coomassie brilliant blue staining. The expression of recombinant protein was confirmed using western blot assay after transferring proteins from polyacrylamide gel onto polyvinylidene difluoride membranes using 2% BSA as a blocking solution, anti-poly-histidine antibody (Sigma, USA) and DAB colored substrate^34^.

### Purification and validation of recombinant peptide

The recombinant peptide extract derived from the periplasmic space injected into the Ni2 + -NTA affinity column (Qiagen Inc., Valencia, CA). The column was washed with 20 mM cold imidazole buffer solution contains 50 mM NaH2PO4, 300 mM NaCl, and 20 mM imidazole. Target proteins were eluted with 250 mM cold buffer imidazole solution. The concentration of purified protein was assessed by Bradford method^32,33^ and high-performance liquid chromatography system (HPLC; Agilent 1260) analysis using C18 reversed-phase column (4.6 × 150 mm, 3.5 μm; Waters, Milford, MA, USA) at 214 nm. Analyses were performed at 40 C for 30 min using a linear gradient of 20 mM sodium phosphate buffer as the mobile phases and solvent mixture of 0.1% Trifluoroacetic in 100% Acetonitrile and 0.1% Trifluoroacetic in 100% Water (35:65 v/v) at a flow rate of 1.0 mL/min^7^. The synthetic buforin I used as standard, the amino acid sequence of buforin I (NCBI accession number: P55897 and amino acid sequence: AGRGKQGGKVRAKAKTRSSRAGLQFPVGRVHRLLRKGNY) then synthesized by the Mimotopes Company (Australia). One mg of buforin I was dissolved in one mL water and dimethyl sulfoxide (DMSO) solution (80:20 v/v) and filter-sterilized (0.22 µm) to prepare a 1000 µg/mL stock solution^6^.

### Investigation of the characteristics of recombinant buforin I peptide

#### Investigation of antimicrobial activity

Antimicrobial effect of recombinant buforin I peptide for *S. aurous* (ATCC 25923), *E. coli* O157: H7 (ATCC 35150), Methicillin-resistant *S. aureus* (ATCC 33591), *L. monocytogenes* (PTCC 1297), *P. aeruginosa*(PTCC 1707), *K. pneumonia* (ATCC 13882), *C. perfringens (*ATCC 13124), *S. Typhi*(PTCC 1609), *A. niger* (PTCC 5010), and *C. albican* (PTCC 5027) were examined. Microbial strains were incubated for 18 to 24 h at optimum strain temperature until reaching half McFarland concentration. The minimum concentration of growth inhibitor (MIC) was determined using the standard sequential dilution method. 90 μl of the prepared concentrations with 10 μl of microbial suspension was incubated in a 96-well plate for 24 h at 37 °C for bacterial strains and 48 h at 25 °C for fungal strains for MIC determination. To determine the MIC, the absorbance at 630 nm (OD_630_) was measured by an ELISA reader. The lowest concentration at which no microorganism growth was observed was reported as the minimum concentration of MIC growth inhibitor. No inoculated growth medium was used as a negative control^35^.

#### Hemolytic and cytotoxicity activities

To prepare the red blood cells, 4 ml of fresh human blood (from volunteer donors) was first centrifuged in 50 μl of EDTA for 10 min at 2422 g. The precipitate was dissolved in 4 ml of PBS buffer and the resulting solution was centrifuged at 1024 g for 10 min. 190 μl of the prepared blood suspension was poured into 7 μl of 1.5 ml and 10 μl of concentrations of 25, 50, 100, 200, 400 μg/ml peptide was added to 5 μL. 10 μl of PBS buffer was added to a microtube as a negative control and 10 μl of Triton X100 added to another tube as a positive control. All microtubes were placed in an incubator at 37 °C. After 30 min, the samples were centrifuged at 1919 g for 5 min. 100 μL of the supernatant from the centrifuge was taken, and its volume was reduced to a final volume of 1 ml by PBS buffer, and then the absorbance of the samples was measured using a spectrophotometer at 570 nm. The uptake of all blood cells treated with serial concentrations of peptide was measured by spectrophotometer and compared with the uptake of a sample containing Triton, and the percentage of hemolysis was calculated.

$${\text{Hemolysis}}\;{\text{percentage}} = \left[ {\left( {{\text{A}}0 - {\text{A1}}00} \right)/\left( {{\text{A}}0 - {\text{AS}}} \right)} \right] \times {1}00$$

As sample adsorption, Ac is negative control sample adsorption and A100 is also positive control sample adsorption. A minimum hemolytic concentration (MHC) was defined as the highest concentration of peptide that does not cause hemolysis^36^.Cytotoxicity evaluated by using HSF (Human Splenic Fibroblasts) cell line DMEM medium containing 1% penicillin and streptomycin and 10% FBS by ATTC culture were maintained. The cells were incubated at 37 °C at 95% humidity and 5% carbon dioxide^37^. The cells were treated in 96-well platelets at concentrations of 0, 250, 500, 1000, and 1500 μg/ml peptide and incubated for another 72 h. Cell viability was assessed by resazurin dye reduction method and by adsorption at 640 nm. Cell viability was calculated using the following formula:

$${\text{Survival}}\;{\text{percentage}} = \left[ {\left( {{\text{A}}_{0} - {\text{A}}_{{\text{C}}} } \right)/\left( {{\text{A}}_{0} - {\text{A}}_{{\text{S}}} } \right)} \right] \times {1}00$$

As adsorption of the sample, Ac adsorption of the control sample (DMEM medium, dye and cell) and A_0_ also adsorption of blank (dye alone)^38^.

### Calculation of therapeutic index

The ratio of MHC to the geometric mean (GM) of MIC against tested microbial strains was used for evaluating the therapeutic index of recombinant buforin I. To calculate the therapeutic index, the two-fold tested concentration (800 g/mL) was employed when no hemolytic activity was detected at the highest concentration tested (400 g/mL)^39^.

### In silico investigation of physical and chemical features peptide modeling

ProtParam (https://web.expasy.org/cgi-bin/protparam/protparam) was applied for predicting physical and chemical features inclouding amino acid composition, molecular weight, theoretical PI, instability index, aliphatic index, amino acid composition, and grand average of hydropathicity (GRAVY) of peptides. Protein-Sol software was used for predicting proteins solubility^11^.

### Peptide modeling, in silico stability investigation and molecular electrostatic potentials

For in silico investigation of stability, the amino acid sequence of buforin I and recombinant buforin I, were modeled with I-TASSER server (https://zhanggroup.org/cgi-bin/itasser) using default parameters^40^. The quality of the models was examined with VADAR server (http://vadar.wishartlab.com/)^41^. For analysis of electrostatic potentials of predicted peptides, PyMol plug-in APBS tools were applied, and PDB2PQR and default grid set-tings were applied for the calculations^31^. The stability of peptides were analyzed using molecular dynamics simulation with the GROMACS 2016.1 package and GROMOS54a7 force field^40^ was solvated in a cubic box using the Simple Point Charge (SPC) water model, for neutralizing the overall charge of the systems, Na^+^ and Cl^-^ ions were added as appropriate. The system was first energy minimized using the steepest descent algorithm to relax high-energy contacts. After energy minimization, the system was simulated under the NPT ensemble for 150 ns, with initial velocities taken from a Maxwell Boltzmann distribution corresponding to 100 K. During this initial simulation time, the peptide was positionally restrained at the temperature was 310 K at 1 atm. The dynamics and stability of peptides under, including root mean square deviation (RMSD), root mean square fluctuations (RMSF), and radius of gyration (RG) were analyzed during the simulation using GROMACS built-in tools^40^.

### In vitro peptide stability investigation

The infrared spectra of individual His-tag buforin I and native buforin I peptides were obtained using a FTIR spectrometer (Thermo Nicolet, AVATAR 370) with an AquaSpec flow-through transmission cell and operating at 4 cm^-1^ spectral resolution^42^. The X-ray diffraction (XRD) patterns of the samples were obtained using an X-ray diffractometer (Explorer, Italy) operating in reflection mode with CuKa (λ = 1.54 A°) radiation at a scan rate of 0.02 to 2 s^−1^. For both experiments the samples were prepared in DMSO (100 mM) and allowed to dry in a freeze dryer (Labconco, USA) before analysis^16^.

### Statistical analysis

In order to confirm the results, the experiments were repeated three times. Results of the study were analyzed by Minitab version 18.0 and differences among the means were determined by one-way ANOVA for significance at *p* < 0.05.

### Ethical approval and informed consent

All experiments were performed in accordance with relevant guidelines and regulations by the clinical studies supervision organization of Iran. All experimental protocols were approved by Ethics Committee of the Ferdowsi University of Mashhad. Written informed consent was obtained from all subjects and/or their legal guardian(s).

## Supplementary Information

Supplementary Information.

## Data Availability

All data generated or analyzed during this study are included in this published article.
