# Supplementary material for: Effects of adding poly-histidine tag on stability, antimicrobial activity and safety of recombinant buforin I expressed in periplasmic space of Escherichia coli
Source: Sci Rep. 2023 Apr 4;13:5508. doi: 10.1038/s41598-023-32782-3 (PMC10073254; doi:10.1038/s41598-023-32782-3)
Supplement: Supplementary file 1 — Supplementary Information. [file 41598_2023_32782_MOESM1_ESM.docx]

**Supplementary figures**


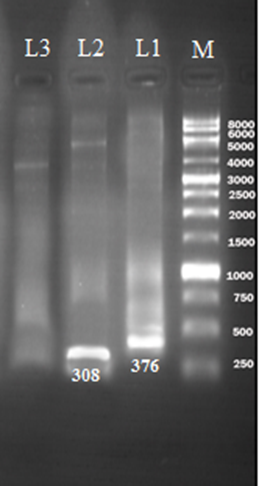


Figure 1: Colony PCR analysis of the recombinant plasmid constructs from *E. coli* DH5 α; M: 1 kbp DNA Ladder, L1: PCR product of *E. coli* DH5α harboring recombinant plasmid pET22 b (+)-BUF, L2: PCR product of *E. coli* DH5α harboring an intact plasmid pET22 b (+) (positive control), L3: PCR product of Plasmid-free *E. coli* DH5α (negative control).


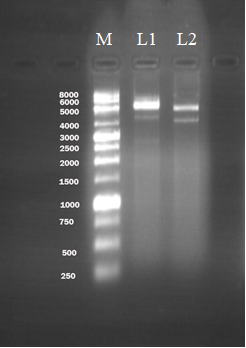


Figure 2: Agarose gel electrophoresis of recombinant plasmid pET22 b (+)-BUF and plasmid pET 22b (+); M: 1 kb DNA ladder, L1: pET22 b (+) -BUF (5554 bp), L2: pET22b (+) (5493 bp).


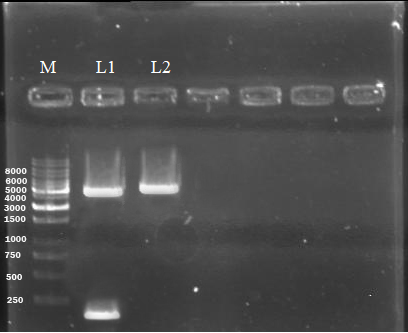


Figure 3: Agarose gel electrophoresis of enzymatic digestion of pET22 b (+)-BUF and native plasmid pET 22b; M: 1 kb DNA ladder, L1: digested recombinant plasmid pET22b (+) -Buforin I with *Nco*I and *Xho*I enzymes, L2: not digested Plasmid pET22b (+).


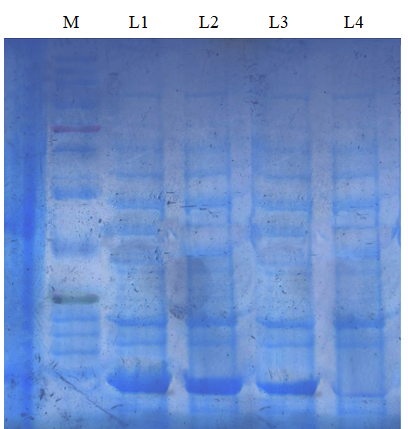


Figure 4: Profile of the expressed buforin I in *E. coli* BL21 (DE3) cells at different sampling times; M) protein ladder, L1) 6 hours, L2) 4 hours, L3) 2 hours, L4) *E. coli* BL21 lacking recombinant plasmid (negative control).


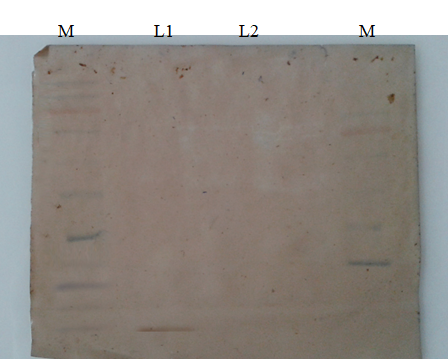


Figure 5: Western blot analysis of the buforin I; M) protein ladder, L1) *E.* *coli* BL21 cell harboring the plasmid pET22b (+)-BUF, L2) *E. coli* BL21 harboring an intact pET22b (+) vector as the negative control.

**
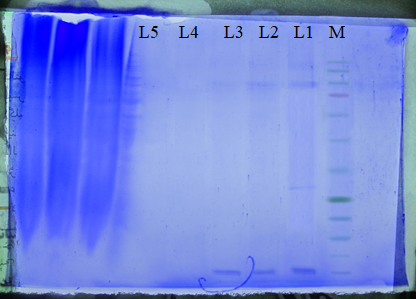
**

Figure 6: SDS-PAGE of purified recombinant buforin I using Ni-NTA column: M) protein marker, L1) Elution 1, L2) Elution 2, L3) Elution 3, L4) Elution 4 and L5) negative control (*E. coli* BL21 without recombinant plasmid).
